# A face sombria da desinformação

**DOI:** 10.1590/0102-311XPT217825

**Published:** 2026-02-16

**Authors:** Romeu Gomes

**Affiliations:** 1 Instituto Nacional de Saúde da Mulher, da Criança e do Adolescente Fernandes Figueira, Fundação Oswaldo Cruz, Rio de Janeiro, Brasil.

A narrativa em questão textualiza um triste capítulo da saúde pública brasileira que assombrou tanto profissionais da saúde, quanto leigos. Nela, são postos em cena dois polos de tensão: a COVID-19 e a desinformação. Ambos puderam e podem não só comprometer a vida, como interrompê-la. A narrativa desses dois polos caminha - ao longo do livro de Neyson Freire ^1^ - num estilo simples e, em determinados trechos, chega a ser didático. Didático no sentido de trabalhar a informação contra a desinformação e de, sobretudo, contar uma história que serve de alerta para que não só se cuide do processo saúde-doença, como também da informação sobre esse processo. Certamente, para alguns acadêmicos, o texto poderia ser mais denso ou analítico. Mas se caminharmos na sua leitura, vamos perceber que o que está em jogo é o estabelecimento de uma comunicação direta sobre o grave problema da desinformação em saúde para um público amplo. Nesse sentido, o livro tem o potencial de ultrapassar um circuito restrito à academia para alcançar um amplo universo de leitores.

O autor é graduado em Tecnologia em Gestão Pública, mestre em Enfermagem, pesquisador e doutorando em Ciências. Ele não só testemunhou a sua história, como também atuou na linha de frente no combate à desinformação sobre a COVID-19.

O cenário em que figuram personagens e enredo na história é o campo profissional de saúde em meio ao drama pandêmico da COVID-19. Esse campo e o seu entorno - entendidos como um espaço (físico ou simbólico) de tensionamento de forças opostas e de luta, onde agentes participavam aceitando regras, impondo-as ou blefando sobre elas ^2^ ^,^ ^3^ - configuraram-se num ambiente polarizado. De um lado, se instaurou um reino de mentira, com imposição de regras e blefes por meio da desinformação e, de outro, armou-se uma resistência à realidade paralela com uma ancoragem nos fatos da pandemia e nos conhecimentos científicos validados. Frente ao cenário dramático de 2020, o autor se desloca do seu estudo acadêmico acerca de competências para conselhos de enfermagem para tomar como seu objeto de investigação o fenômeno das fake news, tentando entendê-lo e, ao mesmo tempo, atuando na linha de frente do combate a esse problema.

Dois personagens centrais se destacam no livro: a desinformação e os profissionais de saúde. A desinformação - entendida como uma informação falsa ou imprecisa com a intenção deliberada de enganar ^4^ - é tratada pelo autor tanto como um conceito imbricado com a infodemia quanto como um fator de presença insidiosa no enfrentamento da pandemia em questão. Esse personagem assume uma característica beligerante que foi não só endossada, como também disseminada pelo governo central brasileiro da época. Nessa época, a desinformação da COVID-19 passa a ser travestida de verdade, uma mentira que não quer ser reconhecida como tal, atendendo a interesses escusos.

O segundo personagem são os profissionais de saúde, sobretudo os da área da enfermagem. No texto, Neyson Freire consegue trazer toda a dramaticidade vivenciada por esses profissionais. O trabalho, que para eles já era difícil e penoso, se agravou na pandemia em questão ^5^. O livro é um tributo aos profissionais que se arriscaram, se estressaram e, alguns, vieram a óbito. Por outro lado, a narrativa também é sobre segmentos dos profissionais de saúde que se alinharam ao principal mandante do reino da mentira, ressignificando a desinformação como informação científica e até mesmo propagando-a. O autor não aborda esse alinhamento de forma geral. Coloca o foco na atuação de um enfermeiro específico, ilustrando como alguém que se forma pela ciência pode não só negá-la, mas também propagar a não ciência.

O enredo da narrativa se inicia pelo susto causado pelo surgimento da pandemia. Nesse ambiente assustador, inicialmente, o foco recai sobre a invisibilidade dos trabalhadores de saúde e a visibilidade das vítimas. Em seguida, se reflete acerca da amplificação da desinformação no país, com apoio de um texto de Maria Helena Machado. No capítulo seguinte, o autor coloca em cena o caso emblemático de um enfermeiro causador da desinformação e vítima do que foi propagado por ele. O terceiro capítulo trata sobre o fenômeno da infodemia. Para contrabalançar o exercício analítico desse fenômeno, o autor nos apresenta - utilizando a via da emoção - uma poesia/crônica de Flávio Liffeman. No capítulo 4, o foco é o problema da hesitação vacinal. Em auxílio a essa discussão o autor nos traz um texto de Isabel Cunha e Luciano Lourenção sobre ameaças à democracia, ciência e saúde mental. Por último, no capítulo 5, o autor discute acerca do futuro da desinformação.

O cenário, o reino da mentira e os personagens (desinformação e profissionais de saúde) abordados no enredo de Neyson Freire nos provocam a manter uma constante vigília sobre a desinformação.

O 1º de abril, tradicionalmente conhecido como o Dia da Mentira, parece ter sido prolongado. Não há mais um só dia atravessado pela mentira. Todos os dias, podemos ser atingidos por ela. As mentiras que circulam em textos ou imagens de forma rápida, independentemente de sua intencionalidade, podem trazer sérias consequências, indo do comprometimento físico e mental das pessoas ao tensionamento social que pode resultar em caos. Nesse sentido, sua face sombria - desinformação travestida de verdade - pode levá-la a ser vista como um gravíssimo problema de saúde pública. Assim, não só o vírus da desinformação deve ser colocado como foco das ações públicas de saúde, como também a promoção de “*competências infocomunicacionais, por meio das quais as pessoas são capazes de lidar com um cenário complexo em que a informação necessária pode estar registrada, mas também pode estar com pessoas*” ^6^ (p. 191).
